# The effectiveness of the use of a digital activity coaching system in addition to a two-week home-based exercise program in patients after total knee arthroplasty: study protocol for a randomized controlled trial

**DOI:** 10.1186/s12891-017-1647-5

**Published:** 2017-07-05

**Authors:** Karen E. M. Harmelink, A. V. C. M. Zeegers, Thijs M. Tönis, Wim Hullegie, Maria W. G Nijhuis-van der Sanden, J Bart Staal

**Affiliations:** 1 FysioHolland Twente, Geessinkbrink 7, 7544 CW Enschede, the Netherlands; 2 Radboud Institute for Health Sciences, IQ Healthcare, Radboud University Medical Center, Nijmegen, the Netherlands; 3 Medisch Spectrum Twente (MST), Koningsplein 1, 7512 KZ Enschede, the Netherlands; 4 Roessingh Research & Development (RRD), Telemedicine group, Roessinghsbleekweg 33b, 7522 AH Enschede, the Netherlands; 5 Faculty of Electrical Engineering, Mathematics and Computer Science, Telemedicine group, University of Twente, P.O. Box 217, 7500 AE Enschede, the Netherlands; 6 Fysiotherapie Hullegie & Richter, Geessinkbrink 7, 7544 CW Enschede, the Netherlands; 7 Faculty of Health and Social Studies, Research Group Musculoskeletal Rehabilitation, HAN University of Applied Sciences, Nijmegen, the Netherlands

**Keywords:** Accelerometer, Home-based exercise program, Total knee arthroplasty, Physical therapy

## Abstract

**Background:**

There is consistent evidence that supervised programs are not superior to home-based programs after total knee arthroplasty (TKA), especially in patients without complications. Home-based exercise programs are effective, but we hypothesize that their effectiveness can be improved by increasing the adherence to physical therapy advice to reach an adequate exercise level during the program and thereafter. Our hypothesis is that an activity coaching system (accelerometer-based activity sensor), alongside a home-based exercise program, will increase adherence to exercises and the activity level, thereby improving physical functioning and recovery. The objective of this study is to determine the effectiveness of an activity coaching system in addition to a home-based exercise program after a TKA compared to only the home-based exercise program with physical functioning as outcome.

**Methods:**

This study is a single-blind randomized controlled trial. Both the intervention (*n* = 55) and the control group (*n* = 55) receive a two-week home-based exercise program, and the intervention group receives an additional activity coaching system. This is a hand-held electronic device together with an app on a smartphone providing information and advice on exercise behavior during the day. The primary outcome is physical functioning, measured with the Timed Up and Go test (TUG) after two weeks, six weeks and three months. Secondary outcomes are 1) adherence to the activity level (activity diary); 2) physical functioning, measured with the 2-Minute Walk Test (2MWT) and the Knee Osteoarthritis Outcome Score; 3) quality of life (SF-36); 4) healthcare use up to one year postoperatively and 5) cost-effectiveness. Data are collected preoperatively, three days, two and six weeks, three months and one year postoperatively.

**Discussion:**

The strengths of the study are the use of both performance-based tests and self-reported questionnaires and the personalized tailored program after TKA given by specialized physical therapists. Its weakness is the lack of blinding of the participants to treatment allocation. Outcomes are generalizable to uncomplicated patients as defined in the inclusion criteria.

**Trial registration:**

The trial is registered in the Dutch Trial Register (www.trialregister.nl, NTR 5109) (March 22, 2015).

## Background

An increasing number of people have some degree of knee pain and functional limitations associated with radiographic evidence of osteoarthritis. Osteoarthritis is associated with pain, stiffness and impairments in activities of daily living [1]. When conservative treatment options, like physical therapy, losing weight, nonsteroidal anti-inflammatory drugs (NSAIDs) and intra-articular injections, are not effective anymore, a total knee arthroplasty (TKA) may be indicated [2]. The number of TKA procedures performed annually is expected to increase due to demographic and anthropometric factors like the ageing population and the increasing incidence of obesity [3]. In the Netherlands, for instance, with a population of 17 million, the number of TKA procedures was about 24,000 in 2013 whereas this figure is expected to rise to 58,000 per year in 2030 [3]. Postoperative physical therapy is the usual care after TKA [4], but there is inconsistent evidence about the effectiveness of physical therapy after TKA [5, 6]. A recent systematic review showed that physical therapy is not very effective in the long term after a TKA in terms of physical function and pain [5]. However, the physical therapy interventions as described in this review consisted of a variety of physical therapy programs such as pool- and gym-based exercises, walking skills and additional balance training or ergometer cycling. However, this review did not study the potentially important role of treatment adherence and did not include studies about home-based exercise programs after TKA. A systematic review by Coppola [7] specifically focused on a comparison between physical therapy and unsupervised home exercise in post-surgical knee disorders. Ten randomized controlled trials (RCTs) were included in this review. In a young and healthy population without comorbidities, supervised physical therapy is not more beneficial than a home-based exercise program following relatively simple knee surgical procedures (such as arthroscopic meniscectomy). There is also consistent evidence that supervised programs are not superior to home-based programs in uncomplicated patients after TKA [8–13]. Important success factors for home-based programs include the adequate patients with a favourable prognosis and increasing the adherence to the program, for instance by telerehabilitation. Tousignant et al. [12] compared the effectiveness of home telerehabilitation with conventional rehabilitation following TKA in a randomized controlled trial. In this study, home telerehabilitation seems at least as effective as conventional rehabilitation. We hypothesize that not all patients will benefit from home-based exercise programs because patients with a worse prognosis need more adapted exercise protocols and hands-on physical therapy support. Over the last few years prognostic factors in relation to TKA have been studied. The risk factors associated with worse outcome are older age [14, 15], obesity [16], worse preoperative physical status [17, 18], more comorbidities [17, 19], lack of self-efficacy [20] and psychological distress [20, 21]. Patients with a good prognosis (active coping and better preoperative functional status measured with the Timed Up and Go test (TUG) and 6-Minute Walk Test (6MWT) [22] probably do not need intensive care from the physical therapists. Possibly, these patients will benefit from a home-based exercise program only. However, adherence to such a program seems pivotal in terms of having an impact. Improvement in pain relief and clinical outcomes in patients with osteoarthritis is largely dependent on intervention adherence [23]. Exercise adherence rates are low in patients with osteoarthritis and arthritis, varying from 53.2 [24] to 70% [25]. Adherence rates were generally higher in supervised programs [26]. Adherence may be improved by an accelerometer-based activity sensor, which provides feedback on the activity level (type of activities performed and the time spent on it). To increase the adherence and thereby improve recovery after TKA we aim to study the effect of the additional use of such an activity coaching system alongside a home-based exercise program. This activity coaching system showed positive results in a pilot study of chronic obstructive pulmonary disease (COPD) patients [27]. The patients were randomly assigned to the activity coaching system or usual care group, and those in the activity coaching system group showed a satisfactory adherence to exercises [27]. We hypothesize that the activity coaching system in combination with the home-based exercise program leads to better adherence and thereby better results in terms of physical functioning than the home-based exercise program in the period of two weeks to three months after TKA surgery.

### Objective

The primary objective of this study is to determine the additive effect of a digital activity coaching system being introduced alongside a home-based exercise program after a TKA on physical functioning measured with the TUG after two weeks, six weeks and three months. The secondary objectives are to determine the additive effect of a digital activity coaching system alongside a home-based exercise program on 1) adherence to the activity level (activity diary); 2) physical functioning measured with the 2-Minute Walk Test (2MWT) and self-reported physical function (Knee Osteoarthritis Outcome Score [KOOS]) at two and six weeks, three months and one year; 3) quality of life (SF-36) at two and six weeks, three months and one year; 4) healthcare use for one year after TKA and 5) cost-effectiveness.

Alongside the RCT, a process evaluation is conducted to determine the usability of the digital activity coaching system in patients after a TKA: a quantitative comparison between the activity diary and the accelerometer data, and interviews to gain insight into the experiences of patients.

## Methods

### Study design

**Fig. 1:**
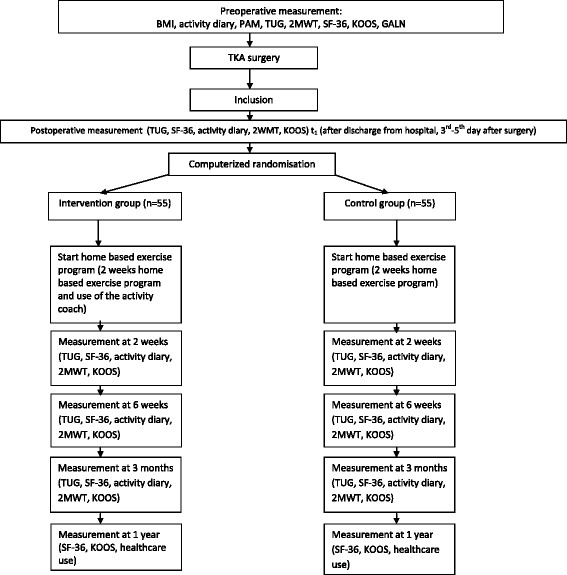
Flow chart

The study design is a single-blinded parallel superiority RCT. The flow chart is shown in Fig. 1. Postoperative measurements take place at the beginning of the home-based exercise program, after two weeks, after six weeks, after three months and after one year. The outcome measures are performed by one researcher, who is blinded to the group assignment.

### Participants

The population consists of patients receiving a TKA with relatively favourable prognosis. In this study we include patients with a good prognosis for functional recovery after TKA to guarantee that patients are able to perform an intensive two-week home-based exercise program on their own. The inclusion criteria are: 1) an adequate preoperative physical status: Bade et al. [21] showed that individuals walking <314 m on the 6-Minute Walk Test (6MWT) before surgery had the poorest physical function six months after surgery. Therefore, we include patients who can walk at least 120 m preoperatively in a 2MWT; 2) absence of compensatory movements during walking, because patients with inadequate walking patterns need more guidance from a physical therapist to relearn a normal dynamic gait (‘no’ on all 13 items of the Gait Analysis List Nijmegen (GALN); [25–27] 3) Body Mass Index (BMI) < 30, because patients with a BMI ≥ 30 kg/m are at higher risk of lower functional outcomes and of complications following primary TKA; [28] 4) age < 80 years because older age is related to worse functioning after TKA; [29] 5) ability to perform activities of daily living independently, which is a requirement for going home after a three-day hospital stay; and 6) having an active coping style as measured with the Patient Activation Measure (PAM) > 60 points. The PAM consists of 22 items. Each item is scored on a four-point Likert scale resulting in a total score range of 22–88. If patients score > 60 points [28], we hypothesize that they are able to take responsibility for their active behavior and adherence to the exercise program.

The exclusion criteria are: 1) all patients with comorbidities (such as heart or lung diseases, (orthopedic) problems in other joints, cancer and movement disorders) who need individually adapted exercise protocols; 2) severe mental disorders; 3) postoperative complications (such as open wounds, infections); 4) delay in recovery shown as >3 days staying in the hospital. All patients were recruited from the Medisch Spectrum Twente (MST) community hospital in the Netherlands.

### Procedures and informed consent

All eligible patients are informed about this study and are invited to participate by the orthopedic surgeon before surgery. The surgeon informs the researcher about patients possibly willing to participate. The researcher informs the patients by phone about the study and provides them with a patient information letter. Patients have one week to consider participating in the study. Patients who are willing to participate sign an informed consent form. After that, the researcher performs the preoperative measures and determines whether the patient is eligible. To minimize the patients’ load, the preoperative assessment is used as an inclusion criterion and preoperative measurement using overlapping tests. Five orthopedic surgeons perform the TKA surgeries. The number of TKA surgeries per year carried out by each surgeon varies from 50 to 70. After surgery, the definitive inclusion takes place if patients are going home after three days post-surgery and no complications are present. After the definitive inclusion, the baseline measurement and randomization take place. The baseline measurement is planned preferably for the third day, and at the latest for the fifth day. A flow chart is shown in Fig. 1. Subjects can leave the study at any time for any reason if they wish to do so without any consequences. The researcher will report serious adverse events to the accredited medical ethical review board of the Medisch Spectrum Twente community hospital in Enschede, the Netherlands, which approved the trial (registration number P15–09, NL52370.044.15). The researcher can decide to withdraw a patient during the study for urgent medical reasons, such as thrombosis, infections of the knee or other complications. KH is the coordinator of the trial and the other authors comprise the steering committee.

### Randomization, blinding and treatment allocation

The orthopedic surgeon checks the inclusion criteria after surgery for definitive inclusion. To assign participants to either of the groups a randomization list is generated by a computer (www.randomization.com) using block randomization with a block size of 6. Pre-stratification is applied for the level of physical activity determined with the activity coaching system prior to surgery (< the median level or ≥ the median level). The median activity level was determined in a pilot of 10 consecutive patients. An independent secretary not involved in enrolling participants in the study prepares concealed, consecutively numbered, sealed, opaque envelopes. The envelopes contain papers indicating the allocated treatment. Participants will receive their envelope at the start of the home-based exercise program from another independent secretary who is not aware of the randomization sequence. Participants can open the envelope while with the physical therapist. Subsequently, the physical therapist will inform the trial coordinator (KH) about the treatment allocation. Patients are instructed not to tell the researcher which group they are randomized to during the follow-up measurements.

### Home-based exercise program

Patients in the intervention group and control group both receive a home-based exercise program. A trained physical therapist experienced in the treatment of patients after TKA and trained in the home-based exercise program explains the program to the patients. There are at least two contact moments: at the start of the program, to give exercise instructions and feedback on the correct performance, and at the end of the program, to monitor the effectiveness. When in need of help the patient is allowed to consult the therapist. The program is explained face to face at the patients’ home immediately after their hospital stay and the exercises are practiced with the patient to be sure that they are done correctly. The program consists of cycling on a home trainer, outside walking and some exercises to improve range of motion (ROM) and muscle strength. The program is shown in Table 1. The home-based exercise program is followed for two weeks and patients are asked to perform the exercises at least five times a day with a gradual increase in time and speed within the two weeks. Patients receive a home trainer for two weeks to do their cycling exercises. Apart from the home-based exercise program patients are allowed to do extra walks or extra daily activities if they want and are able to do so. They are instructed to avoid strenuous activities such as playing football or other sports activities*.* Patients are informed that they can contact the physical therapist if they need consultation and are allowed to receive other kinds of care (e.g. help in the household). There is no limit on the number of consultations needed, because patients need to be sure that they do their exercise adequately. Healthcare use (including physical therapy consultations) is compared between the intervention and the control group.

**Table 1:** exercise program

Week 1	
Day 1	• Explaining home-based exercise program through physical therapist
	• All exercises were explained
Day 2	• Cycling on a hometrainer 2 × 10 min
	• Walking outside 2 × 15 min
	• RoM exercises knee 15 min
Day 3	• Cycling on a hometrainer 2 × 10 min
	• Muscle strength exercises quadriceps 15 min
	• Walking up and down stairs 5 min
	• Walking outside 15 min
Day 4	• Hometrainer 1 × 10 min and 1 × 15 min
	• Walking outside 2 × 15 min
	• RoM exercises knee 15 min
	• Muscle strength exercises quadriceps 15 min
Day 5	• Hometrainer 1 × 10 min en 1 × 15 min • Walking outside 2 × 15 min
	• RoM exercises knee 15 min
	• Muscle strength exercises quadriceps 15 min
Day 6	• Hometrainer 2 × 15 min
	• Walking outside 2 × 15 min
	• RoM exercises knee 15 min
	• Muscle strength exercises quadriceps10 minutes
Day 7	• Hometrainer 2 × 15 min
	• Walking outside 1 × 20 min
	• RoM exercises knee 10 min
	• Muscle strength exercises quadriceps 15 min
Week 2	
Day 8	• Hometrainer 2 × 15 min
	• Walking outside 2 × 20 min
	• RoM exercises knee 15 min
	• Muscle strength exercises quadriceps 15 min
	• Walking up and down stairs 5 min
Day 9	• Hometrainer 2 × 15 min
	• Walking outside 2 × 20 min
	• RoM exercises knee 15 min
	• Muscle strength exercises quadriceps 15 min
Day 10	• Hometrainer 2 × 15 min
	• Walking outside 2 × 20 min
	• RoM exercises knee 15 min
	• Muscle strength exercises quadriceps 15 min
Day 11	• Hometrainer 2 × 20 min
	• Walking outside 1× 30 min
	• RoM exercises knee 15 min
	• Muscle strength exercises quadriceps 15 min
Day 12	• Hometrainer 2 × 20 min
	• Walking outside 1× 30 min
	• RoM exercises knee 15 min
	• Muscle strength exercises quadriceps 15 min
Day 13	• Hometrainer 2 × 20 min
	• Walking outside 2 × 30 min
	• RoM exercises knee 15 min
	• Muscle strength exercises quadriceps 15 min
Day 14	• Hometrainer 2 × 20 min
	• Walking outside 2 × 30 min
	• RoM exercises knee 15 min
	• Muscle strength exercises quadriceps 15 min

### Activity coaching system in the intervention group

**Fig. 2:**
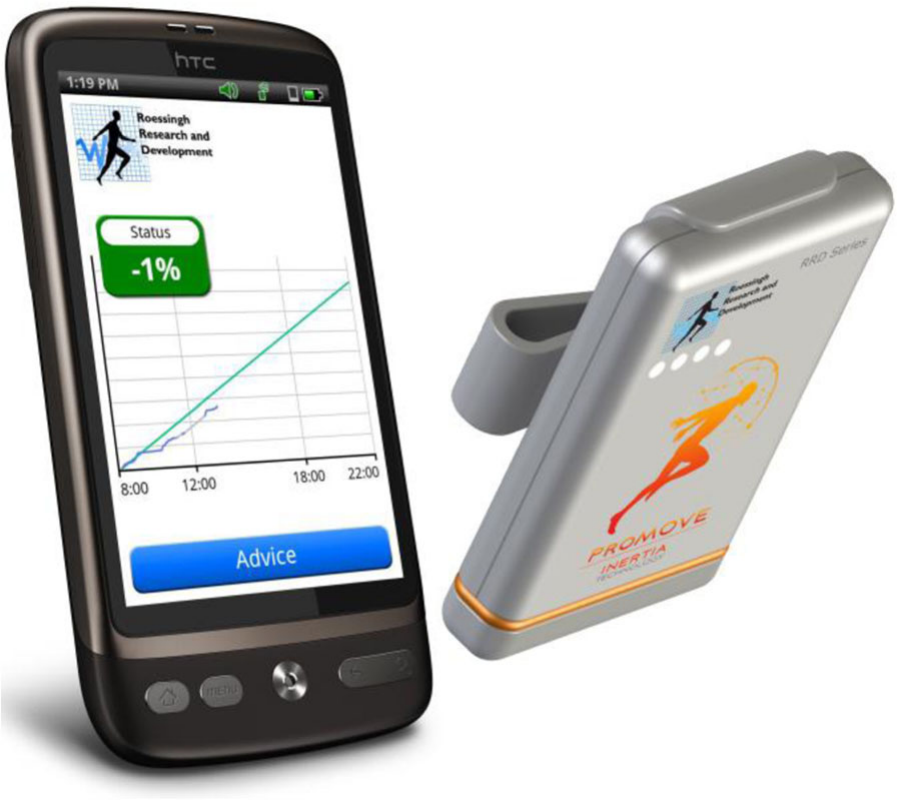
Activity coaching system, consisting of a smartphone and an accelerometer based activity sensor

The home-based program is exactly the same for the intervention and the control group. The only difference is the addition of the digital activity coaching system in the intervention group. The intervention group wears the activity coaching system during the home-based exercise program for two weeks: After two weeks’ use, the influence of the activity coaching system seems to diminish because patients include exercises in daily routines as shown in patients with COPD [27]. Physical therapy after TKA is effective when it starts early [29], so we hypothesize that adding the activity coaching system in the first two weeks [30, 31] stimulates people towards a more active and healthy activity behaviour. It consists of an accelerometer-based activity sensor (Promove 3D, Inertia Technology, Enschede, the Netherlands), which is worn on the patient’s hip combined with a smartphone (Desire S; HTC, New Taipei, Taiwan). The activity coaching system is shown in Fig. 2 and consisted of a tri-axial piezoelectric accelerometer. The accelerometer measures accelerations in the anteroposterior, mediolateral and longitudinal axis of the trunk. The acceleration is band pass filtered, with cut-off frequencies at 0.11 and 20 Hz as described by Bouten et al. [32] and is used to remove the gravitational component from the accelerometer signals. These filtered signals are used to calculate the Integral of the Modulus of the Accelerometer output (IMA) values for the most recent 60 s. The IMA values as measure for physical activity are expressed as mean acceleration per minute and defined as counts per minute [32].

The smartphone application shows two lines: the activity line (in counts per minute) and the reference activity line (in counts per minute). The activity line (displayed on the screen as a blue line) shows the distribution of activities during the day, the intensity of the activities and the duration of the activities. The reference activity line is the cumulative activity the patient should aim for (displayed on the screen as a green line). The reference activity line represents an even distribution of activity intensity during the day. The cumulative activity level represented by this reference line is based on the average cumulative activity level of a previously recorded group of healthy subjects [33].

Patients receive visual feedback in a graph displaying the cumulative reference activity line together with the activity line of the patient drawn minute by minute. Besides that the system coaches towards a healthy activity pattern by providing time-based motivational cues in the form of messages on a smartphone during the day for creating both awareness (how active was the patient up to that moment in time) and extra motivation (remember messages such as go cycling, walking or doing exercises). In addition, text messages contain advice on how to use the affected leg (e.g. symmetric load during transfers and walking). Feedback messages are based on the activity level of the last two hours and of the day so far. They provide a summary of the activity behaviour and advice on how to continue it, i.e. to do more or less to gain their reference activity level.

### Baseline characteristics of participants

Evaluation of baseline characteristics provides insight into the generalizability of the study, the success of the randomization and any potential confounders. Baseline characteristics of participants collected preoperatively are: age (years), gender, BMI, activity level (activity diary), coping style (PAM), walking pattern (GALN), physical status (TUG, 2MWT, KOOS) and quality of life (SF-36). This is shown in the flow chart (Fig. 1).

### Primary outcome

The primary outcome is the between-group difference in physical functioning as measured by the TUG after two weeks, six weeks and three months. The TUG measures the time it takes a patient to rise from an armchair (seat height of 46 cm), walk 3 m, turn, and return to sitting in the same chair without physical assistance [34]. Patients are instructed to walk safely, but as fast as they can. This test has excellent interrater (ICC 0.97) and intrarater reliability (ICC 0.96), as measured in a group of 65 subjects (aged 45–70) with knee osteoarthritis [34]. The TUG has also been shown to be responsive to change after TKA [35]. The TUG is recorded twice and the highest score of the two measurements is analyzed.

### Secondary outcomes

Secondary outcomes are divided into adherence to the activity level (activity diary), performance-based physical function (2MWT), self-reported physical function (KOOS), quality of life (SF-36) and healthcare use.

- Adherence to the activity level measured with the activity diary

Activity level was defined as the type of activities performed and the time spent on it. Because of the absence of a valid and reliable activity-level questionnaire for patients after a TKA an activity diary was developed. The activity diary focuses on physical activities and there is low burden to administer. Every day patients report which activities they have performed and for how many minutes. Walking, cycling, exercises, walking up and down stairs, household chores, pastime and leisure activities, work and sports activities are mentioned in the activity diary and the patient has the opportunity to note other activities and the time spent on them. Other authors used similar activity diaries in healthy adults [36–38]. The activity diary must be filled out one week preoperatively, during the home-based exercise program, in the sixth week, in the third month and one year after surgery (during one week). The minutes spent on each type of activity will be compared to the recommended time per activity as defined in the home-based exercise program (Table 1) to calculate the percentage of adherence (this can be lower or higher than 100%).

- Performance-based physical function measured with the 2MWT

The 2MWT measures walking speed. The outcome is the distance in meters. The 2MWT record on a smooth floor with sufficient walking space over a distance of 10 m. The measurements take place at the patients’ home and therefore 10 m was determined as standard because this is the maximum available space at home. The patients have to walk as many meters as possible in two minutes. They are allowed to use a walking aid [39]. The 2MWT is performed after two and six weeks and three months. During the preoperative 2MWT the Gait Analysis List Nijmegen is used to measure the quality of the gait pattern.

- Self-reported physical function, measured with the Knee Osteoarthritis Outcome Score (KOOS)

The KOOS assesses function over the previous week and is composed of five subscales: pain, symptoms, activities in daily living, activities in sports and leisure, and knee-related quality of life [40–43]. Answers are given using a Likert scale ranging from 0 to 4. A normalized score (100 indicates no symptoms, and 0 indicates extreme symptoms) is calculated for each subscale. The KOOS has been shown to have excellent reliability and good content and construct validity when used for short- and long-term follow-up of knee injury [42, 43]. It has been validated for people with TKA and has been used to evaluate physical therapy outcomes [42, 43]. The KOOS is scored after two and six weeks, three months and one year.

- Quality of life (QoL), measured with the Short Form 36 (SF-36)

The SF-36 is a multipurpose, short-form health survey with 36 questions that measure eight domains of QoL (vitality, physical functioning, bodily pain, general health perceptions, physical role functioning, emotional role functioning, social role functioning and mental health). It has been shown to capture improvements in seven of its eight domains in patients after TKA in the first three months after surgery [44] and continues to indicate improvements in health-related QoL over the next six months [45]. In addition, the results of the eight domains have been combined into two summary scores: physical component score and mental component score [46]. The SF-36 is scored after two weeks, six weeks, three months and one year postoperatively.

- Healthcare use

Healthcare use includes all kinds of healthcare consumed by the patient in relation to TKA surgery beginning at the start of the home-based exercise program up to one year postoperatively. It includes physical therapy, hospital visits, visits of the general practitioner and medication use. The patient sign informed consent to ask for an overview of healthcare costs (health insurance, physical therapist) and patients report how much pain medication (paracetamol, NSAIDs) they use for their knee pain. The use of pain medication is asked about through a medication diary. Healthcare use is measured during the period up to one year after surgery.

- Adherence to the home-based exercise program (intervention group) measured with the accelerometer (activity coaching system)

The accelerometer is only used by patients in the intervention group. The 3D accelerometer is the sensor used by the activity coaching system to register activity levels [27]. The outcome of the accelerometer data are outlined above and shown in Fig. 2. The activity level per day in counts per minute (final point of the blue line in Fig. 2) will be compared to the reference activity line per day in counts per minute (final point of the green line) to calculate the percentage of adherence (this can be lower or higher than 100%). Wearing the accelerometer (adherence to the accelerometer) is determined by asking the patient to report the time of the start and end of wearing the activity coaching system on a form. This is checked with the data on the activity coaching system. The accelerometer and the activity diary are both assessments of physical activity [47].

### Process evaluation

We used a mixed-methods approach in the process evaluation: a quantitative comparison between the activity diary (type and duration of activities performed) and the accelerometer data, and interviews to gain insight into the experiences of patients.

### Interviews

Every third patient who receives the activity coaching system will be asked to participate in a qualitative semi-structured interview to determine the usability of the activity coaching system. Semi-structured interviews with the patients will be conducted three months after surgery. The interviews will take place at the patients’ home and the duration of the interview is at most 30 min. The interviews will be conducted by an independent researcher not involved in the measurements or treatments to guarantee independence. Inclusion of patients will be stopped if saturation is reached (no new information in the last three interviews).

### Sample size

The sample size is based on the primary outcome measure TUG. The sample size is determined using a longitudinal regression model with three follow-up measurement points (two and six weeks and three months). According to the formula described by Twisk [48] for longitudinal studies with repeated measures the required sample size is calculated assuming a mean effect of 10% difference for the TUG scores between the groups over time and a standard deviation of 4.2. These parameter values are based on a study by Kennedy et al. on the clinimetric properties of the TUG in patients with knee and hip arthroplasty [35]. We further assume a correlation coefficient of 0.4 for the correlation between the follow-up measurements. Assuming a dropout rate of 15% a total number of 110 patients is needed. The recruitment period is from 1 April 2015 to 1 August 2017.

### Statistical analysis

#### Patient characteristics

Data will be analyzed with the IBM Statistical Package for the Social Sciences (SPSS) version 22. Patient characteristics at baseline will be analyzed anonymously and compared using measures of central tendency and dispersion (mean, standard deviation, median and range) for continuous variables and percentages for dichotomous variables.

#### Primary and secondary outcomes

The analyses will be performed following the intention-to-treat principle. Between-group differences of the primary outcome measure (TUG) measured at baseline, two weeks, six weeks and three months will be analyzed using generalized estimating equations (GEE) with an exchangeable correlation structure. GEE is a longitudinal data analysis technique that is suitable for investigating the course over time of the outcome variable and comparing this overall effect between study arms [49, 50]. Because GEE can adequately handle missing values (<20%), no imputation technique is planned on beforehand.

The continuous secondary outcomes adherence to the activity level, 2MWT, KOOS, SF-36 and healthcare use will be analyzed in the same way using GEE as well.

Differences in the baseline measurements and baseline characteristics of the two groups could potentially act as confounders. Only when the regression coefficient of the intervention variable in the GEE model changes by at least 10% after adding these variables to the model will they be adjusted for in the analyses [48]. A *p* < 0.05 is considered statistically significant. Patients cannot be deprived from other kinds of care and exercises. Therefore, other types of care and exercises are registered and reported.

#### Healthcare costs and cost-effectiveness

The total healthcare costs will be determined after one year and include all costs related to the TKA surgery measured by the healthcare insurance. The total costs are measured as a continuous variable over a period of 12 months. The cost-effectiveness will be analyzed based on group differences in healthcare costs (number of consultations and other healthcare use). The time off work or caregiver time off work is not measured. This economic analysis is based on the principles of a cost-utility analysis. The primary outcome measures for the economic evaluation are costs and quality-adjusted life years measured with the SF-36. The incremental cost-effectiveness ratio (ICER) “cost per quality-adjusted life years (QALY) gained” will be calculated. Uncertainty surrounding this ICER will be determined using a nonparametric bootstrap method. A cost-effectiveness acceptability curve will be extracted. The impact of uncertainty surrounding parameters of the ICER will be explored using one-way sensitivity analysis on the range of extremes.

In the quantitative process analysis we will calculate the correlation between the data registered in the activity diary (performed activities and the time spent on it) with the data from the activity coaching system and the usability of the activity coaching system using semi-structured interviews. The audio-records from the interviews will be transcribed verbatim and checked for accuracy by KH. Thereafter the transcripts will be analyzed using qualitative data analysis with an open-coding system [51]. Afterwards a thematic analysis will be used to get insight in the experiences of the participants with the program, the impact of the activity coach on adherence and the possible barriers and facilitators. These data will be used to generate possible hypotheses on mediating factors which can be tested using the quantitative data [52].

## Discussion

This study aims to evaluate the (cost-)effectiveness of an activity coaching system in addition to a home-based exercise program using an RCT design. This is one of the first RCTs aimed at optimizing a home-based exercise program by increasing the adherence to physical therapy exercise advice to reach an adequate exercise level during the program and thereafter. Moreover, in this study the patients are allowed to consult the physical therapist if they need to. The home-based exercise program is specifically tailored for patients after TKA and is given by specialized physical therapists with experience in the treatment of TKA. The strength of this study is that both self-reported questionnaires and performance-based tests are used. Mizner et al. concluded that functional improvement after TKA should be measured with both performance-based and patient-reported measurements [53]. We think that the two-week home-based exercise program is particularly appropriate for patients with a reasonably good prognosis for recovery after TKA (see the inclusion criteria) based on the literature and our own clinical practice. This study is generalizable to patients after TKA with a favorable prognosis (better preoperative physical status and preoperatively independent in activities of daily living).

Although we expect positive results from both interventions, we expect 10% more effect on physical functioning measured with the TUG in the group receiving the activity coaching system. This is an estimated effect [35]. It is not clear whether patients seek additional healthcare during the home-based exercise program. Possibly, less healthcare utilization is an effect of the intervention. The ideal study protocol is the addition of a third group, namely no care, to evaluate natural recovery. This was considered not possible and not ethical because physical therapy after TKA is regular in the Netherlands.

## Abbreviations

2MWT: 2-Minute Walk Test
6MWT: 6-Minute Walk Test
BMI: Body mass index
COPD: Chronic obstructive pulmonary disease
GALN: Gait Analysis List Nijmegen
GEE: Generalized estimating equations
ICC: Intraclass correlation
ICER: Incremental cost-effectiveness ratio
IMA: Sum of the integrals of the modulus of each axis of the filtered 3D accelerometer
KOOS: Knee Osteoarthritis and Outcome Score
NSAID: Nonsteroidal anti-inflammatory drugs
PAL: Physical activity level
PAM: Patient Activation Measure
QALY: Cost per quality-adjusted life years
QoL: Quality of life
RCT: Randomized controlled trial
ROM: Range of motion
SF-36: Short-Form 36
SPSS: Statistical Package for the Social Sciences
TKA: Total knee arthroplasty
TUG: Timed Up and Go
VAS: Visual Analogue Scale
WOMAC: Western Ontario and McMaster Universities Arthritis Index
